# Delayed presentation of retrocecal appendicitis in a low-resource setting causing necrotizing lumbar infection and spontaneous colocutaneous fistula: a case report

**DOI:** 10.1093/jscr/rjag719

**Published:** 2026-08-14

**Authors:** James Alex Sumawe

**Affiliations:** Department of Radiology, University of Dar es Salaam, Mbeya College of Health and Allied Sciences, Mbeya, Tanzania; Department of Radiology, Mbeya Zonal Refferal Hospital, Mbeya, Tanzania

**Keywords:** retrocecal appendicitis, necrotizing soft tissue infection, colocutaneous fistula, case report, delayed presentation

## Abstract

Retrocecal appendicitis can present atypically, resulting in delayed diagnosis and potentially severe complications. I report a 43-year-old man who developed a foul-smelling right lumbar wound following two weeks of abdominal pain and swelling. Abdominal computed tomography revealed extensive necrotizing soft tissue infection secondary to a retrocecal inflammatory process, complicated by spontaneous colocutaneous fistula formation. Exploratory laparotomy revealed cecal perforation at the appendiceal base with posterior abdominal wall extension. Right hemicolectomy, ileotransverse anastomosis, lavage, and debridement were performed. Histopathology showed acute and chronic inflammation without malignancy. This case underscores the critical role of early imaging and prompt surgical management in atypical appendicitis to prevent severe complications.

## Introduction

Acute appendicitis remains one of the most common causes of acute surgical abdomen worldwide, with a lifetime risk of ~7%–8% [1, 2]. Delayed presentation and atypical clinical manifestations remain important contributors to morbidity from appendicitis, particularly in resource-limited settings where access to early diagnostic imaging and surgical care may be limited [3]. Retrocecal appendicitis is especially challenging to diagnose because its retroperitoneal position may mask classical right iliac fossa signs, leading instead to flank pain, back pain, or non-specific abdominal symptoms [4]. Such delays increase the risk of perforation and retroperitoneal spread of infection.

Perforated appendicitis may result in complications including retroperitoneal abscess, psoas abscess, necrotizing soft tissue infection, and, rarely, spontaneous enterocutaneous or colocutaneous fistula formation [5, 6]. Infection may extend from the retroperitoneum into the posterior abdominal wall through fascial planes, causing extensive soft tissue involvement [7]. Although uncommon in modern practice, these advanced presentations are still encountered in resource-limited settings where delayed diagnosis and treatment remain prevalent [3].

Contrast-enhanced computed tomography (CT) plays a pivotal role in atypical and complicated appendicitis by accurately localizing the appendix, confirming the diagnosis, and identifying associated intra-abdominal complications such as perforation, abscess formation, and peritonitis. Furthermore, CT delineates the extent of retroperitoneal and soft tissue spread, thereby facilitating appropriate surgical planning and management [8].

Management requires prompt resuscitation, broad-spectrum antibiotics, and definitive surgical source control, ranging from appendectomy to right hemicolectomy in cases with extensive cecal involvement or tissue destruction.

## Case presentation

### Patient information and clinical findings

A 43-year-old man presented to the emergency department with a 1-week history of progressively discharging foul-smelling purulent material from a right lumbar wound. This was preceded by a 2-week history of worsening right iliac fossa and right flank pain associated with progressive swelling. He also reported constipation but denied vomiting, abdominal distension, or urinary symptoms. He had no history of diabetes mellitus, tuberculosis, inflammatory bowel disease, malignancy, or previous abdominal surgery. On examination, the patient appeared cachectic but was hemodynamically stable. Local examination revealed an irregular ulcerative lesion over the right lumbar region with a 2 × 2 cm sinus actively discharging purulent material. The surrounding skin was erythematous, warm, and tender to palpation.

### Laboratory and imaging findings

Initial laboratory investigations demonstrated significant leukocytosis (21.12 × 10^9^/L) with neutrophilic predominance (90.4%), consistent with severe bacterial infection. Hemoglobin was reduced at 8.7 g/dl, suggestive of anemia of chronic disease or ongoing inflammatory state. Renal function and electrolytes were within normal limits on admission.

**Figure 1:**
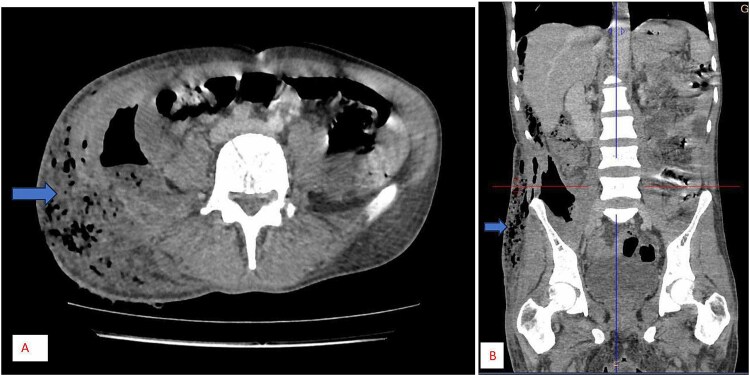
Preoperative contrast-enhanced CT abdomen and pelvic images axial (A) and coronal (B) showing extensive right lumbar necrotizing soft tissue infection with multiple air foci and surrounding inflammatory stranding (arrows).

**Figure 2:**
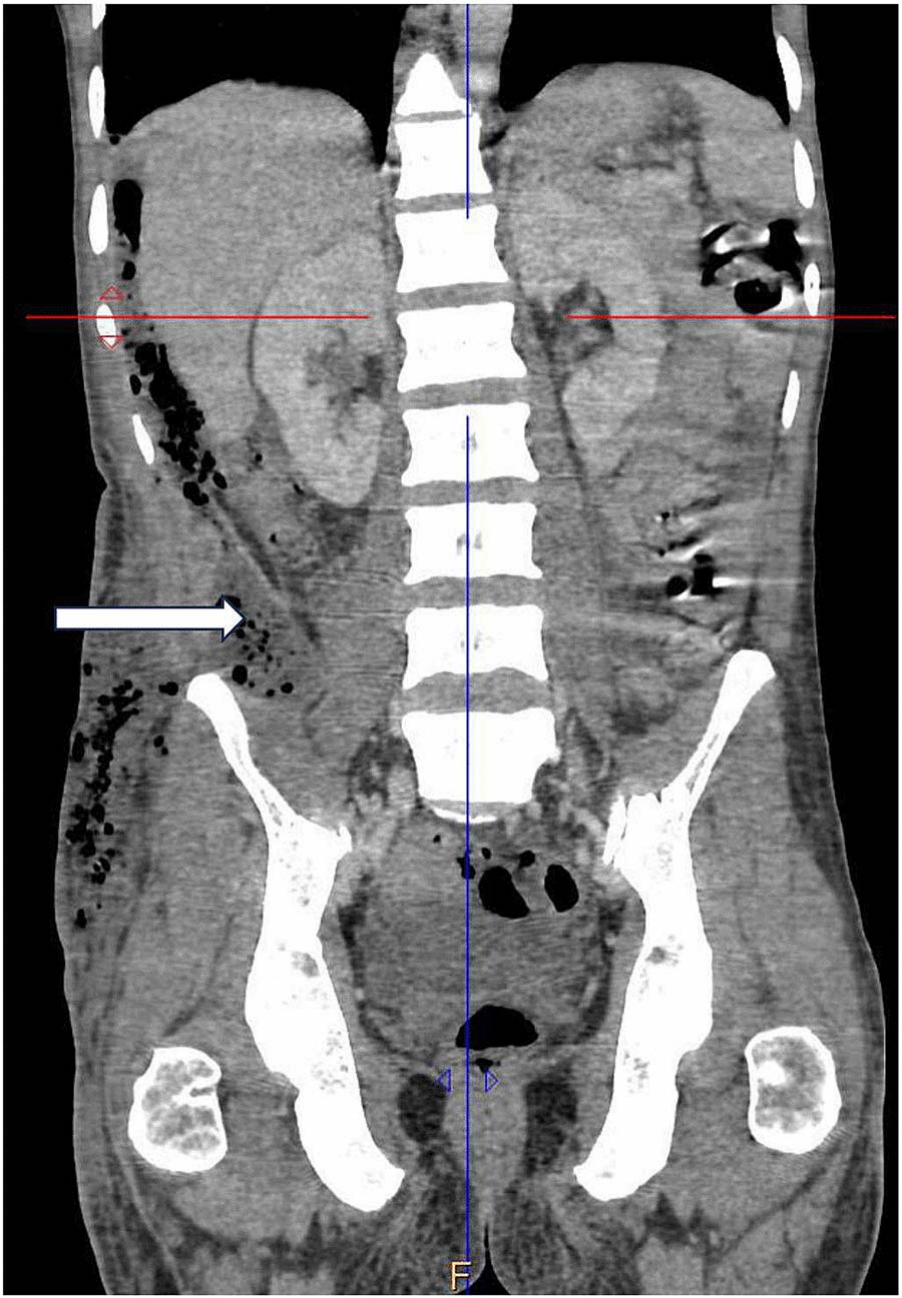
Preoperative contrast-enhanced CT abdomen and pelvic coronal image showing extensive infected collections extending from the retrocecal region into the right paracolic gutter (arrow) and retroperitoneal spaces, with superior extension toward the perihepatic region.

Contrast-enhanced CT of the abdomen and pelvis demonstrated extensive necrotizing soft tissue infection involving the right lumbar subcutaneous tissues and adjacent paraspinal musculature, characterized by multiple gas locules and associated fluid collections, consistent with necrotizing fasciitis (Fig. 1). The inflammatory process extended through the posterior abdominal wall musculature, with a focal defect communicating with the intraperitoneal cavity. Within the abdomen, multiple air-containing fluid collections were identified in the right paracolic gutter, with superior extension toward the right subhepatic/perihepatic region, associated with surrounding fat stranding (Fig. 2). The epicenter of the inflammatory changes was localized to the retrocecal region, with features highly suggestive of perforated retrocecal appendicitis complicated by extensive retroperitoneal and abdominal wall spread.

### Initial management and clinical course

The patient was initially managed as a case of sepsis secondary to ruptured lumbar abscess with suspected intra-abdominal source. Broad-spectrum intravenous antibiotics were initiated, including amoxicillin-clavulanate and metronidazole, later escalated to ceftriaxone-sulbactam and subsequently meropenem due to severity of infection and imaging findings. Supportive care included intravenous fluids, analgesia, correction of electrolyte imbalance, blood transfusion for anemia, and regular wound dressing. Despite initial stabilization, the patient continued to have persistent purulent discharge, raising suspicion of enteric communication and evolving colocutaneous fistula formation.

### Operative findings and surgical management

Exploratory laparotomy via midline incision revealed dense inflammatory adhesions involving the cecum and ascending colon, which were fixed to the right iliac fossa. A perforation was identified at the base of the appendix extending into the adjacent cecum, with retroperitoneal spread of infection tracking through the posterior abdominal wall and communicating with the right lumbar wound. Right hemicolectomy with ileotransverse end-to-side anastomosis was performed, along with thorough peritoneal lavage and debridement of necrotic tissues. The external lumbar wound was irrigated and partially closed with drainage.

### Histopathology and outcome

Histopathological examination demonstrated acute and chronic inflammatory cell infiltrates consistent with severe suppurative inflammation, with no evidence of dysplasia or malignancy. The postoperative course was uneventful. The patient showed gradual clinical improvement, with normalization of leukocyte counts and resolution of systemic inflammatory signs. He was discharged on oral antibiotics and continued with regular follow-up. At 4 months postoperatively, the wound had completely healed, and there was no evidence of recurrent infection or fistula formation.

## Discussion

Retrocecal appendicitis is a diagnostically challenging variant of acute appendicitis due to its retroperitoneal location, often resulting in minimal early peritoneal signs and delayed diagnosis [4]. In the setting of delayed or unrecognized perforation, particularly with a retrocecal appendix, infection may extend beyond the peritoneal cavity into the retroperitoneal spaces. The spread of polymicrobial enteric organisms through these anatomical planes can result in severe complications, including retroperitoneal abscess formation and necrotizing soft tissue infection involving the abdominal wall, flank, or lumbar region [5, 6, 9]. Because the initial clinical manifestations may be dominated by skin and soft tissue findings, such as pain, swelling, erythema, necrosis, or purulent discharge, the underlying intra-abdominal source can be overlooked [9]. This atypical presentation may lead to misdiagnosis as a primary soft tissue infection and delay definitive management of the causative abdominal pathology. Recognition of this potential pathway of disease spread and early use of contrast-enhanced CT are essential for identifying the source of infection, defining the extent of involvement, and guiding appropriate surgical source control [8].

Spontaneous appendicocutaneous fistula is a rare complication of perforated appendicitis. It usually develops following chronic untreated or inadequately treated appendiceal inflammation, where localized perforation leads to abscess formation, adherence of the appendix to the abdominal wall, and gradual maturation of a fistulous tract to the skin. Patients may present with a chronic discharging sinus or feculent cutaneous discharge rather than typical features of acute appendicitis, making diagnosis challenging. Cross-sectional imaging plays an important role in identifying the underlying appendiceal pathology and defining the fistulous tract [7, 8, 10].

Management of complicated appendicitis requires prompt hemodynamic resuscitation, early administration of broad-spectrum antibiotics, and definitive surgical source control [10]. Early surgical intervention remains the cornerstone of treatment in perforated appendicitis and should not be unduly delayed, particularly in patients with extensive local or retroperitoneal disease. The choice of operative procedure depends on the severity and extent of tissue involvement. While appendectomy is adequate in most cases, more extensive resections, including ileocecal resection or right hemicolectomy, may be necessary when there is significant cecal involvement, extensive inflammation, tissue necrosis, or destruction of the appendiceal base that precludes safe primary closure [10]. In patients with severe retroperitoneal extension or necrotizing soft tissue infection, aggressive debridement and adequate drainage are essential components of source control and may require staged surgical procedures. A multidisciplinary approach involving surgeons, radiologists, intensivists, and infectious disease specialists is often necessary to optimize outcomes in these complex presentations [10].

This case highlights that retrocecal appendicitis may initially present as necrotizing lumbar infection or fistula, emphasizing the importance of early CT imaging, high clinical suspicion, and timely surgical intervention to prevent severe complications.

## Conclusion

This case demonstrates the potential severity of delayed retrocecal appendicitis, where concealed perforation may progress to extensive retroperitoneal infection and spontaneous colocutaneous fistulization. A high index of suspicion, early CT evaluation, and prompt multidisciplinary surgical management are essential to achieve favorable outcomes in such uncommon presentations.
